# Impact of an online WHO mental health guideline-based training on stigma and clinical confidence among non-psychiatrist physicians in Mexico: a pre-experimental pilot study

**DOI:** 10.3389/fpubh.2025.1697178

**Published:** 2025-11-05

**Authors:** Ingrid Vargas-Huicochea, Ana Carolina Rodríguez-Machain, Silvia A. Tafoya

**Affiliations:** Department of Psychiatry and Mental Health, Faculty of Medicine, Universidad Nacional Autónoma de México (UNAM), Mexico City, Mexico

**Keywords:** mental health services, social stigma, clinical competence, primary care physicians, continuing medical education

## Abstract

**Introduction:**

Mental health is a critical component of overall well-being, and the stigma associated with mental illness often prevents healthcare professionals from providing adequate care. This is a pilot study designed to evaluate the feasibility of an educational intervention and its preliminary effects on stigma associated with mental illness and the subjective assessment of capacity, clinical experience, and perceived limitations in managing mental disorders among non-psychiatrist physicians.

**Methods:**

A pre-experimental design was used, including measurements before (Pre), immediately after (Post), and Follow-up at 12 months after the intervention (F-12 m). The sample consisted of non-specialist doctors who were invited to a training program of mhGAP and all scheduled assessments. Inclusion criteria were: being an active non-psychiatrist doctor, providing informed consent, and availability to participate in all three phases of the study. The online training program was based on the WHO mhGAP guidelines and it was administered over a period of 5 weeks in three groups in February, March and August 2023. The instruments used were the Mental Illness Clinician Attitudes (MICA) and the Attitudes, Confidence, and Behavior Questionnaire (ARCBQ), which measures capacity, experience, and limitations perceived in managing patients with mental illness.

**Results:**

Of the 69 doctors enrolled, 39 (57%) were drop-outs and 30 (43%) completed the training and all evaluations. The results show a significant decrease in stigma and a notable increase in perceived capacity and clinical experience over time. However, perceived limitations did not change significantly.

**Conclusion:**

The intervention has important implications for physician training and suggests the need for a more inclusive and sustainable approach to continuing mental health education. These findings pave the way for future research on the longitudinal impact of such changes and their applicability across diverse cultural and demographic contexts.

## Introduction

Mental health is an essential component of overall human well-being; it is inextricably linked with physical, social, and emotional activities. An enduring disparity continues, especially in low- and middle-income countries, between the high burden of mental health needs in primary care and the ability of their non-psychiatrist physicians to respond to these needs (1). This gap is not merely a short-lived problem limited only by a lack of resources; the gap is also framed by attitudinal and operational obstacles to clinical practice. A significant number of frontline clinicians experience poor self-efficacy, clinical uncertainty, and negative attitudes toward mental illness which impact the quality and timeliness of care (2–4). Stigma of this kind, personal or institutional, can result in the under-recognition of symptoms, the procrastination to refer to services or, in severe cases, the omission of mental health issues despite prior training (5–7). Importantly, bridging this divide is not just about transferring knowledge.

There is evidence that stigma acts as an attitudinal barrier in decreasing use of mental health services and self-efficacy is described as the professional’s confidence in practicing knowledge in real-world settings, a significant predictor of clinical behavior (8, 9). An examination of these two dimensions gives richer insights into the impact of education on practice beyond educational learning.

Non-psychiatrist providers are therefore a prime target for mental health education. In low- and middle-income settings, up to 90% of people with mental disorders do not receive treatment, and primary care is often their sole point of contact with the health system. General practitioners and other non-specialists are therefore the first – and often the only – line for detection, initial management, and early referral (1, 2). Yet most lack formalized training in mental health and report discomfort when dealing with psychiatric symptoms, which can lead to underdiagnosis, ineffective management, or delayed action (10–12). In conclusion, their technical skills and attitudes need to be strengthened to bridge the treatment gap, facilitate earlier interventions, and promote more humane, effective, and accessible care in health care systems.

The Mental Health Gap Action Programme (mhGAP) 2.0, developed by the World Health Organization (WHO) (13), represents a key strategy for closing this gap through structured training for non-specialist professionals. Their practical and standardized approach to disorders such as depression, anxiety, psychosis, and suicide risk has been shown to improve diagnosis and clinical management in various global contexts (14, 15). Studies in low- and middle-income countries have reported increases in timely detection, better follow-up, and greater user satisfaction after implementation (16, 17), although with limited measurement of objective clinical outcomes in patients (18).

In Mexico, experiences in Tamaulipas, Jalisco, Chiapas, and Mexico City reflect progress in the acceptance and application of mhGAP, highlighting improvements in clinical competence and willingness to provide mental health care (19–22). However, structural challenges remain: resource shortages, work overload, social stigmatization (23, 24), and *ad hoc* training that does not guarantee knowledge acquisition or attitude change (25, 26). Furthermore, there is little evidence on how sociodemographic variables influence the response to these interventions, despite studies suggesting differences in empathy and perception of clinical burden among professionals (27–29).

In light of this training gap, the present study evaluates the impact of an educational intervention based on mhGAP 2.0 on stigma, perceived capacity, and clinical experience in primary care physicians, also analyzing the role of gender and marital status. Using a pre-post quasi-experimental design, we seek to provide useful evidence to strengthen more humane, contextualized, and sustainable mental health training strategies, aimed not only at informing but also at transforming clinical practice.

## Materials and methods

### Participants and procedure

This study used a pre-experimental design (30) with repeated measures at three time points: before the intervention (Pre), immediately after (Post), and at 12 months follow-up (F-12 m), with the aim of assessing changes in stigma, perceived capacity, clinical experience, and self-imposed limitations in non-psychiatrist doctors after an educational intervention based on the WHO mhGAP 2.0 guidelines.

The call for participation was disseminated electronically among groups of physicians, and the sampling was based on convenience. The inclusion criteria for the course stipulated that the participants must be licensed general practitioners, non-psychiatrists. Interns who had already completed their general medical training were eligible to participate. The training certificate was provided to participants who filled out the registration form (which included the pre-intervention measurement instruments), completed the 5 weeks of training, and responded to all the instruments in the follow-up evaluations.

The intervention consisted of a 40 h online training program delivered over five consecutive weeks. This training intervention for mental health care was designed to be carried out online by the Research Coordination Unit of the Department of Psychiatry and Mental Health of the UNAM Faculty of Medicine. The sessions were facilitated by two of the study’s authors: a psychiatrist (IVH), certified as a mhGAP trainer by the World Health Organization (WHO), and a clinical psychologist (ACRM), certified by the Pan American Health Organization (PAHO). The third author (SAT), also PAHO-certified as a mhGAP trainer, contributed to the pedagogical design and coordination of asynchronous components. All three facilitators have extensive experience in mental health education and primary care integration. The instructional sessions were conducted in three distinct groups, which were categorized as A, B, and C.

The asynchronous and synchronous activities based on the mhGAP guide, covered the following modules:

IG mhGap 2.0 Essential Mental Health Care and Practices Module.
Depression Module of the mhGap 2.0 IG.
Self-Harm/Suicide Module of the mhGap 2.0 IG.
Psychosis Module of the mhGap 2.0 IG.

The training was conducted over 5 weeks, with 1 week per module, except for the psychosis module, which took 2 weeks because schizophrenia was covered in 1 week and bipolar disorder in the other.

The asynchronous activities for each module were carried out from Monday to Thursday, and constant feedback was provided through the Google Classroom platform designed for this activity. In the classroom, each module consisted of the following activities:

1. Review of literature with information based entirely on mhGAP.
2. Analysis and discussion in a video guide forum that was conducted according to the corresponding mhGAP module, which contained all the clinical, procedural, and therapeutic information that appears in the guide.
3. Analysis and discussion in a forum of a publicly accessible video testimonial obtained from the YouTube platform, which was related to the corresponding module and aimed to raise awareness among participants.

The synchronous activities were carried out on the fifth day of the week, after the asynchronous activities, and lasted 4 h. During this workshop session, the participants worked on:

1. A knowledge assessment through the Kahoot platform, with review and feedback on each question.
2. Analysis of a clinical case together with the psychiatrist from the research team, as well as the presentation and resolution of doubts regarding the condition according to that week’s module.
3. Role-playing exercises to assess questioning and non-specialized management skills, evaluated with a generic rubric for addressing mental health in primary care.

The dates on which the groups were held are as follows:

Group A: February 6, 2023, to March 2, 2023.
Group B: March 6 to March 31, 2023.
Group C: August 7 to September 1, 2023.

The assessment instruments used in this research were applied before the training, immediately after the training, and 12 months after the training was completed.

The final analytical sample consisted of 30 non-specialist physicians who completed both the full five-week online training program and all scheduled assessments at three time points: before the intervention (Pre), immediately after (Post), and 12 months later (F-12 m). Of the 69 physicians who initially enrolled (100%), 36 (52%) completed all training modules. However, only 30 (43% of initial enrollees) fulfilled the dual requirement of finishing the course and providing complete outcome data at all three assessment waves, thus being included in the longitudinal analysis.

Participants were excluded from the main analysis if they either did not complete the training or failed to submit one or more evaluation instruments at any time point. Among the 39 participants classified as dropouts (57% attrition), reasons for non-completion included work commitments (*n* = 27), personal reasons (*n* = 6), and missing at least one assessment (*n* = 6). It is important to clarify that this attrition comprised two distinct groups: first, a substantial proportion (*n* = 33; 48% of total enrollees) registered but never initiated the training—they did not attend any synchronous session or engage with asynchronous materials. This group likely reflects initial interest without follow-through, possibly due to scheduling conflicts or competing demands. Second, among the 36 who began the course, 6 (8.7% of total enrollees) completed all 5 weeks of training but failed to complete one or more assessments and were therefore excluded from the final analysis.

There were no cases of partial completion of the training program itself—i.e., no participant started the five-week intervention and subsequently dropped out before finishing it. All those who initiated the course completed it in full. Therefore, the primary barrier to inclusion in the analysis was not non-completion of the educational content, but rather missing outcome data at one or more assessment points. Qualitative feedback collected at the end of the intervention suggests that challenges such as clinical workload, limited availability, and, in some cases, difficulties navigating digital platforms contributed to non-compliance with follow-up assessments. While the length and timing of the synchronous sessions may have played a role, they were not identified as the sole or primary cause. A secondary qualitative analysis based on focus groups is currently underway to explore perceptions of feasibility, workload, and digital accessibility. These findings will be reported separately.

### Measures

Outcome variables were evaluated using validated, self-administered instruments via a digital platform, ensuring anonymity and confidentiality.

Stigma toward mental illness was measured using the MICA (Mental Illness Clinician Attitudes) scale, which has adequate internal consistency (*α* = 0.72) evaluated in healthcare students and professionals (31). A higher score indicates greater stigma. In our study, the reliability for MICA score was α = 0.57.

Perceived self-efficacy was assessed using three scales of the ARCBQ (Attitudes Reported, Confidence and Behavior Questionnaire) (32), whose components were analyzed separately: ARCBQ-C for perceived capacity, ARCBQ-E for perceived experience, and ARCBQ-L for perceived limitations, allowing a multidimensional assessment of the physician’s subjective experience in clinical practice. A higher score indicated greater capacity, experience, and perceived limitations, in that order. In our study, the reliability score was *α* = 0.86 for ARCBQ-C, α = 0.78 for ARCBQ-E, and α = 0.70 for ARCBQ-L.

The instruments were adapted to Mexican Spanish in a sample different from that of this study, according to the recommendations of Gaite et al. (33), or which a multiphase interactive translation model was used and subsequently the cross-cultural applicability of the instruments was verified through a pilot study in graduate medical.

### Data analysis

Statistical analysis included descriptive statistics to characterize the sample. Since the scores of the study variables did not show a normal distribution (assessed using the Shapiro–Wilk test), non-parametric tests were used. Differences between measurement times (Pre, Post, and F-12 m) were analyzed using the Friedman test with Conover + Holm correction for *post hoc* analysis, and the role of sociodemographic variables was explored by no parametric correlations or comparative tests. The level of significance was set at *p* < 0.05. All analyzes were performed using JASP software version 0.18.3.

### Ethical considerations

The study complied with the ethical principles established in the Declaration of Helsinki. It was approved by the Ethics and Research Committees of the responsible university institution (FM/DI/018/2025), and all participants signed an informed consent form before inclusion. The rights to confidentiality, anonymity of data, and the possibility of withdrawing from the study at any time without affecting participation in the training were guaranteed.

## Results

**Figure 1:**
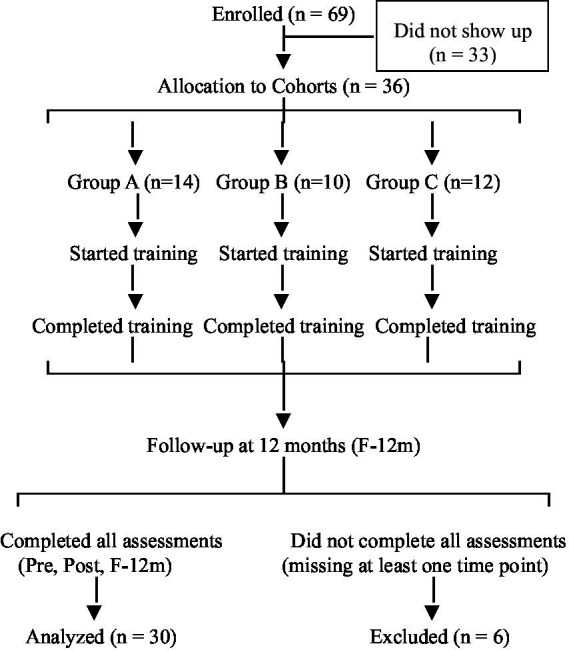
CONSORT-style flow diagram illustrating participant progression through the study phases. Of the 69 individuals who registered for the training, only 30 completed it in accordance with the participation requirements; 33 registered but did not attend the training. Of the 36 who took the five-week training, 30 responded to all follow-up instruments, while 6 missed one or more of the follow-up assessments. Exclusion was due to missing follow-up data, not dropout from the training itself.

The sample consisted of 30 non-specialist doctors who completed the training program and all scheduled assessments. Of an initial total of 69 registrants (100%), 36 (52%) completed the training but only 30 (43%) completed the data collection at all three assessment times (Pre, Post, and F-12 m) and were included in the final analysis (see Figure 1). Among the 33 subjects who registered but did not show up to undergo the intervention, the main reasons for dropping out were work commitments (*n* = 27, 81.8%), and personal reasons (*n* = 6, 18.2%).

A comparison was made between those who completed the training and those who did not. Table 1 shows that those who completed the course were younger and had less professional experience; those who participated in the February cohort had the highest dropout rate.

**Table 1:** Characteristics of the population that started and completed the course.

	Completed the course		χ^2^	*p*
	No (*n* = 39)	Yes (*n* = 30)		
	*n* (%)	*n* (%)		
Sex				
Female	33 (85)	22 (73)		
Male	5 (13)	8 (27)		
Prefer not to say	1 (2)	0 (0)	3.12^*^	0.210
Marital status				
Single	14 (37)	15 (50)		
Married/Commun-law	19 (50)	15 (50)		
Divorced	5 (13)	0 (0)	6.49^*^	**0.039**
Course group				
A	21 (54)	13 (43)		
B	1 (3)	9 (30)		
C	17 (43)	8 (27)	11.40^*^	**0.003**

	Mdn (IQR)	Mdn (IQR)	*U*	*p*
Age (years)	41.4 (10.6)	34.4 (10.0)	800.0	**0.005**
Professional experience (years)	16.5 (12.8)	12.8 (14.0)	794.5	**0.002**
Stigma toward mental illness (MICA initial score)	61.5 (9.0)	60.5 (7.8)	629.5	0.350
Perceived capacity (ARCBQ-C initial score)	9.5 (5.3)	9.0 (3.8)	548.0	0.923
Perceived experience (ARCBQ-E initial score)	9.0 (3.0)	9.0 (2.0)	532.0	0.922
Perceived limitations (ARCBQ-L initial score)	19.0 (8.0)	19.5 (5.0)	539.5	0.849

“Completed the course” refers to participants who finished all five weeks of training. Inclusion in the final analytical sample required both course completion and full participation in Pre, Post, and F-12 m assessments (*n* = 30). Of the 39 classified as “No,” 33 never initiated the training, and 6 started but missed one or more evaluations. Mdn, median; IQR, inter-quartile range; MICA, mental illness clinician attitudes; ARCBQ, attitudes, confidence, and behavior questionnaire; C, capacity; E, experience; L, limitations. * Likelihood Ratio Chi-Square. Statistically significant values are presented in bold.

The final sample consisted of 22 (73%) women and 8 (27%) men, with a mean age of 34.2 ± 7.4 years and a mean professional experience of 8.7 ± 7.7 years. Marital status was evenly distributed, with 15 (50%) participants single and 15 (50%) married or in a common-law relationship. Participant characteristics are shown in Table 1.

An analysis of the sociodemographic characteristics was performed. Age was not correlated with any study variable: Stigma toward Mental Illness (rho = 0.13, *p* = 0.501), Perceived Ability (rho = 0.13, *p* = 0.480), Perceived Experience (rho = −0.05, *p* = 0.802), and Perceived Limitations (rho = −0.07, *p* = 0.727). Similarly, years of professional experience showed no significant association: Stigma (rho = 0.09, *p* = 0.639), Perceived Capacity (rho = 0.08, *p* = 0.668), Perceived Experience (rho = −0.06, *p* = 0.744), and Perceived Limitations (rho = 0.01, *p* = 0.954). The study found no statistically significant differences based on gender or marital status in relation to the examined study variables. Only a trend toward difference was observed in Perceived Limitations by course group, with the A group (χ^2^ = 5.82, *p* = 0.054), see Table 2.

**Table 2:** Effect of participant characteristics on study variables.

	Stigma toward mental illness (MICA initial score)	Perceived		
		Capacity (ARCBQ-C initial score)	Experience (ARCBQ-E initial score)	Limitations (ARCBQ-L initial score)
	Mdn (IQR)	Mdn (IQR)	Mdn (IQR)	Mdn (IQR)
Sex				
Female	59.5 (6.8)	9.0 (3.0)	9.0 (2.0)	19.5 (4.8)
Male	60.0 (10.8)	9.5 (5.0)	10.0 (3.5)	19.5 (5.5)
*U*	116.50	81.50	97.50	86.50
*p*	0.188	0.776	0.668	0.962
Marital status				
Single	61.0 (10.5)	9.0 (3.0)	10.0 (2.5)	20.0 (4.5)
Married/Commun-law	59.0 (6.5)	9.0 (4.5)	8.0 (1.5)	19.0 (5.5)
*U*	140.50	119.00	144.50	129.00
*p*	0.253	0.801	0.184	0.505
Course group				
A	61.0 (6.0)	9.0 (4.0)	8.0 (1.0)	21.0 (5.0)
B	52.0 (10.0)	9.0 (3.0)	9.0 (2.0)	20.0 (4.0)
C	63.5 (8.5)	10.5 (3.8)	10.5 (2.5)	15.5 (4.5)
*χ^2^*	4.75	0.20	3.22	5.82
*p*	0.903	0.904	0.200	0.054

Mdn, median; IQR, inter-quartile range; MICA, mental illness clinician attitudes; ARCBQ, attitudes, confidence, and behavior questionnaire; C, capacity; E, experience; L, limitations.

**Figure 2:**
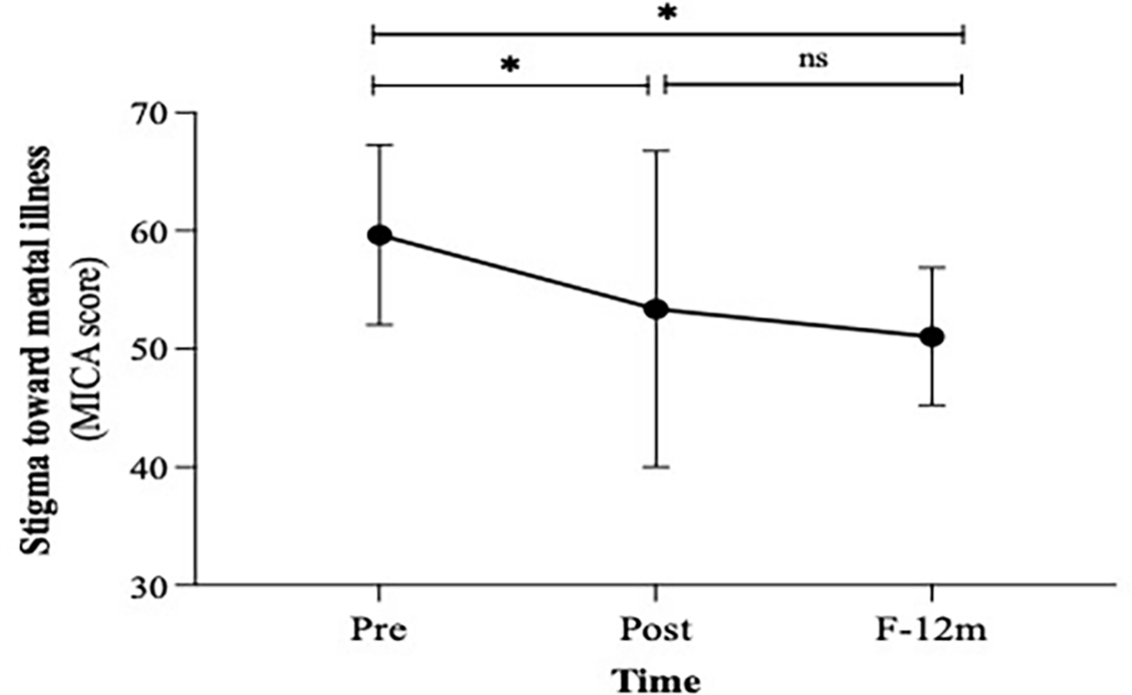
Changes in stigma toward mental illness. F-12 m, Follow-up at 12-month; MICA, mental illness clinician attitudes. * *p* < 0.01, ns = not significant.

The educational intervention based on mhGAP 2.0 showed significant effects in reducing stigma toward mental illness. MICA scores decreased significantly from Pre to Post and remained reduced at F-12 m (χ^2^_F_ = 12.29, *p* = 0.002). No significant differences were observed between Post and F-12 m, indicating stability of the anti-stigma effect over time (Figure 2).

**Figure 3:**
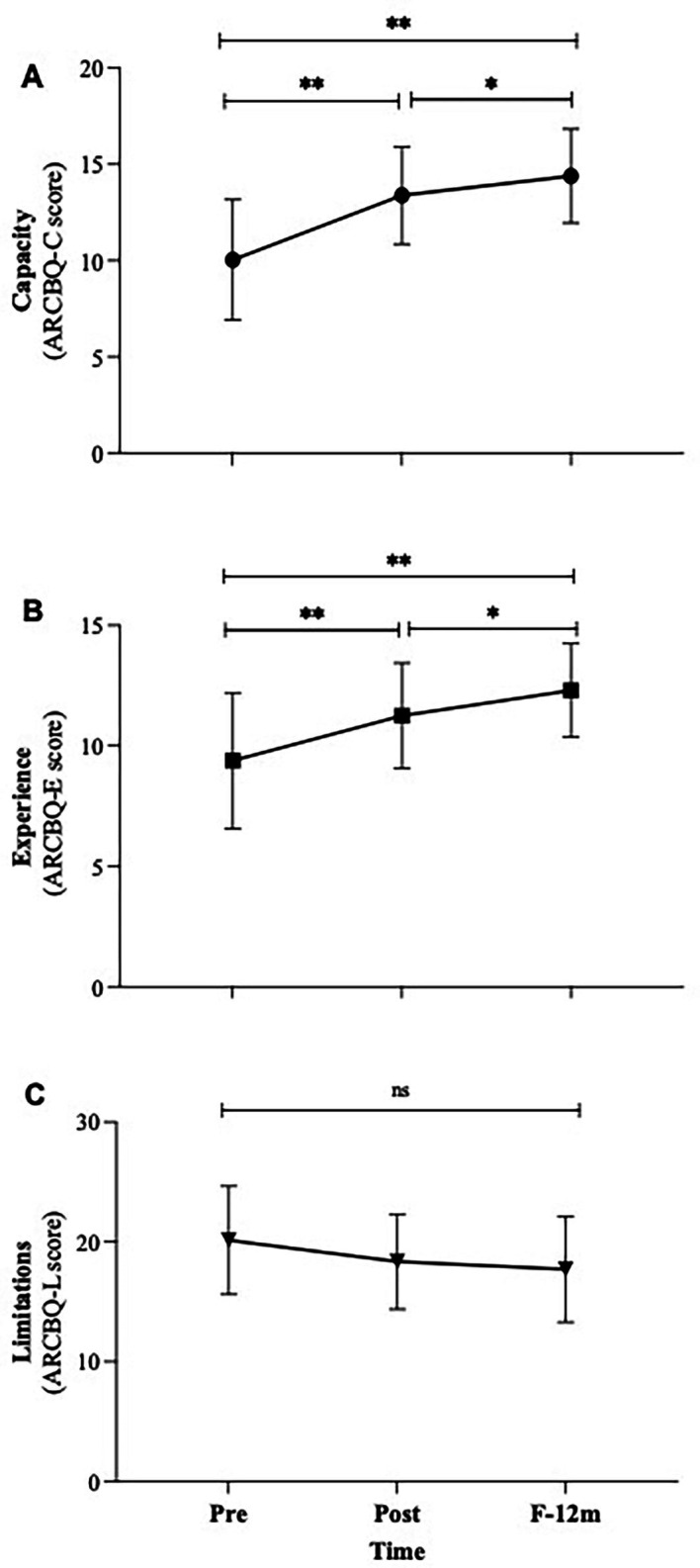
Changes in the perceived capacity **(A)**, experience **(B)** and limitations **(C)** in addressing mental health problems. F-12 m, follow-up at 12-month; ARCBQ, attitudes reported, confidence and behavior questionnaire. * *p* < 0.05, ** *p* < 0.00, ns = not significant.

Self-rated capacity to manage mental health problems increased significantly and was sustained. ARCBQ-C scores rose significantly from Pre to Post and remained high at F-12 m (χ^2^_F_ = 25.23, *p* < 0.001). A further significant increase was observed between Post and F-12 m, suggesting consolidation of professional confidence over time (Figure 3A). Similarly, subjective experience in managing mental health problems increased significantly. ARCBQ-E scores rose from Pre to Post and at 12 months (χ^2^_F_ = 23.47, *p* < 0.001), with an additional significant increase between Post and F-12 m (Figure 3B). Although Perceived Limitations decreased, changes were not statistically significant at any time point (Figure 3C).

## Discussion

This research provides evidence on the impact of an educational intervention based on the WHO mhGAP 2.0 guidelines on non-psychiatrist physicians, aiming not only to impart knowledge but also to raise awareness and facilitate skill acquisition. It is important to note that this study is preliminary, designed primarily to test training materials and assessment tools in a real medical education setting. Although the final sample size is limited and the dropout rate high, the results are consistent, clinically relevant, and provide a solid basis for future research and methodological improvements. Its value lies not only in the magnitude of the observed effects but also in the feasibility of implementing and evaluating structured mental health education strategies within resource-constrained health systems.

An important observation emerging from the data is the significant and sustained reduction in stigma toward mental illness, a well-documented barrier in the international literature that affects both physician willingness to intervene and the quality of care provided (3, 4). The reduction in stigma, as measured by the MICA scale, remained at 12 months, in contrast to prior studies where the impact of educational measures appeared to have been transient (25). This finding is especially relevant in situations like Mexico, where stigmatizing medicalization of mental illness and the invisibility of mental disorders in primary healthcare facilities persist despite numerous training interventions (23, 24). The sustained anti-stigma effect suggests that combining technical content with awareness-raising strategies—such as testimonial videos and role-playing—can generate deeper changes in social representations of mental suffering. This is very well coordinated with the educational package developed by Rezvanifar et al. (34)—who, through Delphi, a panel of experts and a scoping review developed a sequential and multimodal intervention for Iranian medical students, specifically to introduce film-based discussion and structured contact with people with psychiatric disorders and facilitated group reflection on individual and systemic stigma. This work suggest that passive exposure is not enough; stigma reduction requires intentional, interactive encounters that promote both cognitive and affective engagement. Similarly, Zare-Bidaki et al. (35) found that when students engaged in one single session of a virtual reality simulation of psychosis (VRSP), stigma was significantly reduced and empathy and knowledge increased more than just clinical observation. Importantly, their results indicate that even technologically-mediated experiential learning can drive perspective-taking if it is carefully designed to mirror authentic lived experiences without perpetuating stereotypes.

In line with the studies described above, our research findings support the emerging literature that argues that transformational educational theories that balance clinical knowledge, experience-based components (like contact with personal experience) and interaction with lived experience, are more successful in the reduction of professional stigma than traditional didactic paradigms (36). Patient testimonials and role playing could have enhanced perspective taking and emotional engagement in our intervention, and possibly played important roles in the lasting decrease in stigmatizing attitudes at 12 months. This is consistent with transformational education frameworks promoting empathy and humanized care (5, 7), and also focusing on the necessity of a mix of direct education with contact and group discussion on defining stigma and personal experiences in order to establish and solidify attitudes change (34). Of course, without leaving aside that powerful, emotional experiences, however fleeting, can drive significant transformation of perception, if they are situated within a pedagogical context that invites reflection and defies assumption (35). We also find evidence from mhGAP-based interventions in Malawi, Pakistan and Nigeria that, in line with our work, have been effective in reducing stigma and increasing self-efficacy (10, 16, 17).

The lasting impact seen at the 12-month mark suggests that incorporating both a tech-informational technology-based and transformational components (e.g., testimonials, simulations, role plays, virtual simulations) can make a more durable lasting impact as to more effectual, lasting changes than purely informative programs. Collectively, this series of studies provide an important point-of-sight conclusion: reducing professional stigma is not just a matter of sharing knowledge; it is about creating empathic understanding through formal, reflective and humanizing education experiences with other people using structured, reflective and people-directed activities. Likewise, the significant and progressive increase in perceived capacity and clinical experience highlights the strengthening of professional self-efficacy. This finding aligns with global evidence indicating that non-specialist physicians, when receiving standardized and contextualized training, not only improve diagnostic skills but also develop greater confidence in managing mental health problems (14, 15). Notably, these scores increased not only immediately after the intervention but continued to rise during the follow-up year, suggesting a consolidation effect in clinical practice. This evolution may reflect progressive internalization of knowledge, enhanced by real-world clinical experience, reinforcing the importance of integrating training with ongoing support and supervision, as observed in successful programs elsewhere (16, 17).

However, it is striking that perceived limitations did not show significant changes. This presents a paradox: physicians feel more capable and experienced, yet do not perceive fewer barriers. This may reflect a heightened awareness of structural health system limitations—such as work overload, lack of resources, or absence of support networks—that training alone cannot resolve. This is consistent with studies indicating that while educational interventions improve individual competence, their real-world impact depends on organizational and systemic conditions often unaddressed in training programs (23, 26). The persistence of perceived structural limitations after the intervention is consistent with the findings of Movahedi et al. (37), who observed that a lack of clear protocols for managing mental health conditions, coupled with the pressure of care, perpetuates negative perceptions and a sense of therapeutic helplessness, even among well-intentioned physicians. Thus, as physicians gain confidence, they may become more critical of their working conditions—a sign of professional maturity rather than regression. In addition to the above, systemic barriers such as heavy workloads, limited access to psychotropic medications, and inadequate referral systems, have been chronicled extensively in low- and middle-income countries’ health systems (2, 23). Solutions to these problems need more than training; they require a multifaceted approach. These initiatives include ongoing clinical supervision [as shown in STEPCARE (14)], clinical guidelines integration into electronic health records, and advocacy for resources within institutions.

No relationship was found between sociodemographic variables and the analyzed outcomes, including gender. Previous studies have suggested differences in empathy and perceived burden by gender (27, 29), yet in this sample, change trajectories were homogeneous. This may reflect limited statistical power due to the small sample size. Interestingly, it is important to note that participants who completed the study were significantly younger than those who dropped out. Previous studies suggest that older professionals may have more stigmatizing attitudes toward mental disorders, attributable to medical training that has historically been less sensitive to mental health (17, 28, 38). Although we do not have measures of stigma among non-participants, it is plausible that stigmatizing attitudes influenced their decision not to continue, introducing a possible selection bias. Future research should evaluate how factors such as age, work context, or previous experiences with mental health influence attitudes, stigma, the adoption of tools such as those in this training, or the possibility of dropout.

This study underscores the need to rethink medical training as a cross-cutting component of health policy. The integration of mhGAP 2.0 should not be limited to isolated courses but should be embedded within medical curricula, continuing education, and clinical support systems. Furthermore, mental health education should extend beyond knowledge transfer to address the affective, ethical, and social dimensions of care, fostering a more humanized and less hierarchical clinical practice. In this sense, the model evaluated here could inform the development of more sustainable educational strategies, particularly in low- and middle-income countries where specialist shortages make generalist training essential (2, 13). We could suggest integrating mhGAP 2.0 at three levels: (i) undergraduate, as a compulsory module in family medicine; (ii) residencies, with rotations in community mental health; and (iii) continuing education, linking certification to professional reaccreditation, as in Jalisco (20). Of course, this is not an easy or immediate task, as it requires partnerships between universities, ministries of health, and PAHO/WHO.

The intervention is scalable due to its digital format and alignment with national policies. Its sustainability, however, depends on recurring funding, training of local multipliers, and integration into official continuing medical education platforms. Experiences in Chiapas and Tamaulipas show that the active participation of health authorities is key to its institutionalization (19, 21).

It is important to acknowledge the limitations of this study. The high rate of attrition (57%) substantially limits the representativeness of the sample, a concern further exacerbated by the imbalanced demographics of the final participants, who were predominantly younger, less experienced, and female. The length of the course (40 h) may also have contributed to dropout, highlighting the importance of considering program intensity and feasibility in future designs. The pre-experimental design imposes constraints on the ability to establish causal inferences, as the absence of a comparison group prevents attributing improvements solely to the intervention. Reliance on self-reported measures introduces the possibility of social desirability bias, particularly regarding sensitive constructs such as stigma and self-efficacy, which may compromise accuracy. Future research should incorporate objective or observer-rated measures and implement strategies to minimize attrition, such as enhanced participant engagement, flexible scheduling, or incentives. The relatively small sample size and specific demographic profile further limit the external validity of the findings, reducing their applicability to broader populations. Moreover, the lack of change in perceived limitations suggests that systemic barriers, such as resource shortages and excessive workload, remain unresolved. Addressing these structural issues is crucial to complement individual-level training. Regarding the instruments of this study, although the MICA and ARCBQ instruments were adapted into Spanish through a rigorous peer review and pilot testing process, no formal psychometric validation in the Mexican medical population is available. This limits the interpretation of absolute scores, although the observed intra-subject changes provide preliminary evidence on the direction and magnitude of the intervention’s effects. Finally, patient-level clinical outcomes were not assessed, leaving a significant gap regarding the real impact on quality of care, a limitation noted in other mhGAP evaluations (18).

Results of our study also suggest that incorporation of mental health training into continuing medical education would be effective, particularly in specialist settings. But lasting effect will take educational intervention and more structural changes to instill confidence—supervision, institutional support, and better referral systems—to convert increased confidence into effective clinical services. Initiatives that address stigma are found to be more likely to be successful when supported by hospital leadership, from policy change and consideration of mental health as a priority to investment in these programs (34). This indicates that scaling up mhGAP-based interventions requires advocacy at the administrative level with the aim of promoting sustainability and systemic impact.

## Conclusion

This research study highlights the importance of an intervention in education rooted in the WHO mhGAP 2.0 Guidelines for decreasing the stigma against mental disorders, and that it can promote perceptions of clinical competence and expertise among non-psychiatric physicians over a sustained period of up to 12 months. However, perceived structural limitations remain unchanged, which indicates that training does not suffice without organizational backing. These results substantiate the inclusion of mental health education with continuing medical education, especially in the context of limited access to specialists.

## Data Availability

The raw data supporting the conclusions of this article will be made available by the authors, without undue reservation.
